# Effect of Immunosuppression on the Immune Response to SARS-CoV-2 Infection and Vaccination

**DOI:** 10.3390/ijms25105239

**Published:** 2024-05-11

**Authors:** Emma J. Leacy, Jia Wei Teh, Aoife M. O’Rourke, Gareth Brady, Siobhan Gargan, Niall Conlon, Jennifer Scott, Jean Dunne, Thomas Phelan, Matthew D. Griffin, Julie Power, Aoife Mooney, Aifric Naughton, Rachel Kiersey, Mary Gardiner, Caroline O’Brien, Ronan Mullan, Rachael Flood, Michael Clarkson, Liam Townsend, Michelle O’Shaughnessy, Adam H. Dyer, Barry Moran, Jean M. Fletcher, Lina Zgaga, Mark A. Little

**Affiliations:** 1 Trinity Kidney Centre, Trinity Translational Medicine Institute, School of Medicine, Trinity College Dublin, D08 W9RT Dublin, Irelandbradyg1@tcd.ie (G.B.);; 2 Department of Nephrology, Galway University Hospital, H91 YR71 Galway, Ireland; 3 School of Biochemistry and Immunology, Trinity Biomedical Sciences Institute, Trinity College Dublin, D02 R590 Dublin, Ireland; orourka3@tcd.ie (A.M.O.);; 4 Department of Clinical Medicine, School of Medicine, Trinity Translational Medicine Institute, Trinity College Dublin, D08 W9RT Dublin, Ireland; 5 Department of Immunology, St. James’s Hospital, D08 NHY1 Dublin, Irelandjedunne@stjames.ie (J.D.);; 6 Regenerative Medicine Institute (REMEDI) at CÚRAM SFI Research Centre for Medical Devices, School of Medicine, University of Galway, H91 TK33 Galway, Ireland; 7 Vasculitis Ireland Awareness, Belfast & Dublin, Ireland; 8 Department of Rheumatology, Tallaght University Hospital, D24 NR0A Dublin, Ireland; 9 Department of Nephrology, Cork University Hospital, T12 DC4A Cork, Ireland; 10 Department of Infectious Diseases, St. James’s Hospital, D08 NHY1 Dublin, Ireland; 11 Discipline of Medical Gerontology, School of Medicine, Trinity College Dublin, D08 W9RT Dublin, Ireland; 12 Department of Public Health and Primary Care, Institute of Population Health, Trinity College Dublin, D02 PN40 Dublin, Ireland

**Keywords:** COVID-19, SARS-CoV-2, immunosuppression, rituximab, vaccine, immune response

## Abstract

Immunosuppressive treatment in patients with rheumatic diseases can maintain disease remission but also increase risk of infection. Their response to severe acute respiratory syndrome coronavirus 2 (SARS-CoV-2) vaccination is frequently blunted. In this study we evaluated the effect of immunosuppression exposure on humoral and T cell immune responses to SARS-CoV-2 infection and vaccination in two distinct cohorts of patients; one during acute SARS-CoV-2 infection and 3 months later during convalescence, and another prior to SARS-CoV-2 vaccination, with follow up sampling 6 weeks after vaccination. Results were compared between rituximab-exposed (in previous 6 months), immunosuppression-exposed (in previous 3 months), and non-immunosuppressed groups. The immune cell phenotype was defined by flow cytometry and ELISA. Antigen specific T cell responses were estimated using a whole blood stimulation interferon-γ release assay. A focused post-vaccine assessment of rituximab-treated patients using high dimensional spectral cytometry was conducted. Acute SARS-CoV-2 infection was characterised by T cell lymphopenia, and a reduction in NK cells and naïve CD4 and CD8 cells, without any significant differences between immunosuppressed and non-immunosuppressed patient groups. Conversely, activated CD4 and CD8 cell counts increased in non-immunosuppressed patients with acute SARS-CoV-2 infection but this response was blunted in the presence of immunosuppression. In rituximab-treated patients, antigen-specific T cell responses were preserved in SARS-CoV-2 vaccination, but patients were unable to mount an appropriate humoral response.

## 1. Introduction

Patients with rheumatic disease requiring immunosuppressive therapy are at increased risk for severe Coronavirus Disease 2019 (COVID-19). This heightened risk has been attributed to demographic factors, a requirement for immunosuppressive therapies, and the presence of comorbidities, including kidney disease [1]. Immunosuppressive treatment for rheumatic diseases can induce and maintain disease remission but also increases risk of infection [2]. Patients with rheumatic or autoimmune diseases, including those with Anti-Neutrophil Cytoplasmic Antibody (ANCA)-associated vasculitis (AAV), have higher rates of hospitalisation and death from COVID-19 [3]. Thus, vaccination against severe acute respiratory syndrome coronavirus 2 (SARS-CoV-2) is important to prevent severe COVID-19 in patients with rheumatic disease. Severe COVID-19 is defined as dyspnoea, respiratory frequency ≥ 30/min, blood oxygen saturation ≤ 93%, partial pressure of arterial oxygen to fraction of inspired oxygen ratio < 300, and/or lung infiltrates > 50% within 24 to 48 h [4].

The first line of host defence against pathogens, including SARS-CoV-2 infection, is the innate immune system [5]. This cellular response limits viral entry, translation, and replication by inducing an inflammatory response and helps identify and remove infected cells. The innate immune response also aids the development of adaptive immunity, largely mediated via a humoral antibody response [5]. Therefore, in patients who are immunosuppressed, their innate and adaptive immune responses can be inadequate resulting in increased susceptibility to infection, more severe infection, and inadequate antibody response. Conversely, it is possible that the hyperimmune response observed in severe COVID-19 may be abrogated in immunosuppressed patients.

Vaccine response may be blunted in patients with AAV by their immunosuppressive treatments [6,7,8]. The prospective, multi-centre, OCTAVE study examined development of anti-spike antibodies (humoral response) following SARS-CoV-2 vaccination in 2204 immunosuppressed patients, including 35 with AAV. Patients with AAV on rituximab had the highest failure rate to generate an antibody response (21/29, 72%), However, most exhibited a satisfactory T cell response (27/29, 93%). Vaccine type also predicted immune response, with mRNA (BNT162b2) vaccine associated with a higher likelihood of antibody response but a lower likelihood of cellular response, compared to the adenovirus vector (ChAdOx1 nCoV-19) vaccine. However, few patients (5/35) with AAV received the mRNA vaccine in this study, therefore between-group comparisons by vaccine type were not possible [9]. In a second UK prospective cohort study examining antibody response to three or more SARS-CoV-2 vaccine doses, those with small vessel vasculitis were least likely to have a positive antibody response (OR: 0.69, 95% CI 0.55–0.87) [10]. T cell responses and comparisons across vaccine types were not examined in this study. Neither study examined the immune response to acute SARS-CoV-2 infection.

We prospectively evaluated the presence and strength of humoral and cell-mediated immune responses to both acute COVID-19 and to SARS-CoV-2 vaccination in adults who were either exposed or not exposed to immunosuppressive therapy. We hypothesised that the interaction of factors, including patient age, immunosuppression exposure/type, and type of SARS-CoV-2 vaccination (mRNA vs. adenoviral vector), would influence B- and T cell responses both to acute COVID-19 infection and to vaccination. 

## 2. Results 

### 2.1. Participants Characteristics

A total of 182 participants were recruited to this study: 121 in Cohort 1 and 61 in Cohort 2. There were no significant differences in age or sex between any of the relevant groups. In Cohort 1 (Table 1), 19 participants were receiving immunosuppressive treatments at the time of COVID-19 infection, most (84.2%) of whom were non-rituximab-exposed. The median age was 55 and 62 for the immunosuppressed and non-immunosuppressed groups, respectively. Cohort 2 comprised 61 individuals: 28 immunosuppressed AAV, 15 non-immunosuppressed AAV, and 18 HC (Table 2). A total of 25% of immunosuppressed patients were rituximab exposed, and 75% were taking other immunosuppressive medications. Participants were sampled prior to vaccination and 4 weeks post vaccination (IQR 28–36.5 days). Overall, 41.9% received an adenovirus-based vaccine (AstraZeneca, AZD1222), and 58.1% received one of the mRNA-based vaccines (Pfizer/BioNTech, BNT162b2 or Moderna, CX-024414).

### 2.2. Immunosuppression at the Time of Acute COVID-19 Causes Lymphoid and Myeloid Subset Effects That Persist into the Convalescent Period

**Figure 1:**
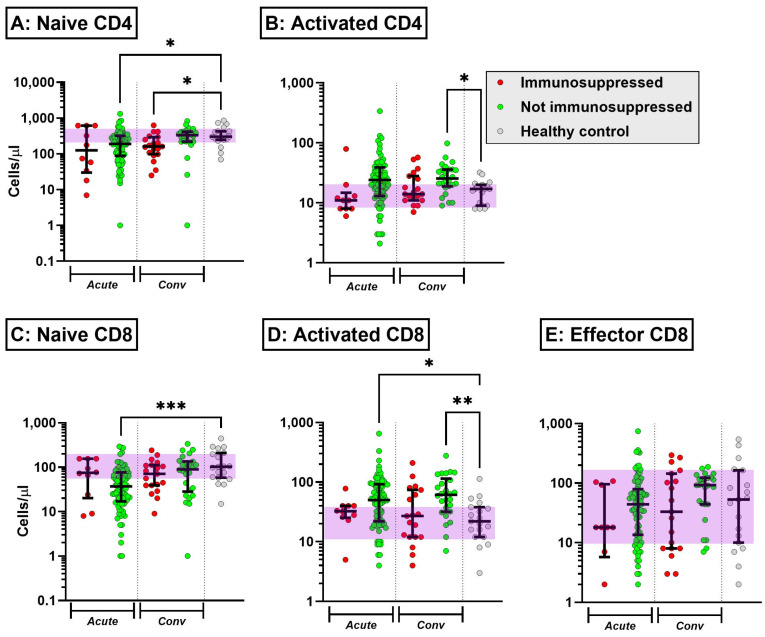
Absolute circulating lymphocyte subset (naïve (**A**) and activated (**B**) CD4+, naïve (**C**) and activated (**D**) CD8+, and (**E**) effector CD8+) counts (median ± interquartile range) in patients with acute COVID-19 (Acute) and at 3 months post infection (Conv), stratified according to the presence of immunosuppression at the time of infection. The purple shading represents the interquartile range of healthy uninfected controls. * *p* < 0.5, ** *p* < 0.005, *** *p* < 0.001, Kruskal–Wallis test with Dunn’s post hoc test comparing individual groups against healthy controls.

**Figure 2:**
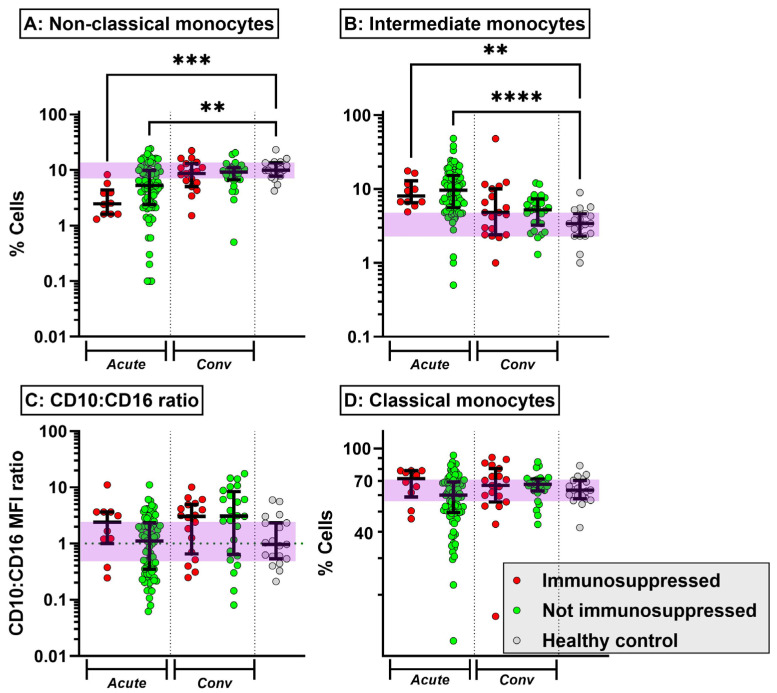
Circulating monocyte subset (non-classical (**A**), intermediate (**B**), and classical (**D**)) fractions (median ± interquartile range) in patients with acute COVID-19 (Acute) and 3 months post infection (Conv), stratified according to the presence of immunosuppression at the time of infection. The CD10:CD16 WFI ratio was also determined on CD15+ neutrophils (**C**). The purple shading represents the interquartile range of healthy uninfected controls. ** *p* < 0.005, *** *p* < 0.001, **** *p* < 0.0001 Kruskal–Wallis test with Dunn’s post hoc test comparing individual groups against healthy controls.

Acute COVID-19 was characterised by T cell cytopenia and reduction in NK cells, which was broadly similar in immunosuppressed and non-immunosuppressed patients (Supplemental Figure S1). Naïve CD4 and CD8 cells were also reduced in acute COVID-19, with the partial abrogation of the drop in naïve CD8 cells in the presence of immunosuppression (Figure 1A,C). Conversely, activated CD4 and CD8 cell counts increased in non-immunosuppressed patients with acute COVID-19, an effect that was reduced in the presence of immunosuppression (Figure 1B,D). The increase in activated CD4 and CD8 T cells persisted into the convalescent period in non-immunosuppressed patients but not in those on immunosuppressive medication. Non-classical monocytes were reduced in acute COVID-19, while intermediate monocytes were increased (Figure 2A,B). Both perturbations resolved in the convalescent period and were not affected by the presence of immunosuppression. There was an increase in immature neutrophils in acute COVID-19. Interestingly, the neutrophil CD10:CD16 ratio was numerically increased in immunosuppressed patients with acute COVID-19, with this observation persisting into convalescence (Figure 2C). The serum levels of interleukin (IL)-6 and sCD25 were elevated in acute COVID-19; this was not influenced by the presence of immunosuppression (Supplemental Figure S2). Thus, exposure to immunosuppression at the time of COVID-19 infection reduces the circulating number of activated T cells but does not prevent shifts in monocyte subsets and neutrophil maturity at the time of acute disease and three months post induction.

### 2.3. Immunosuppression at the Time of SARS-CoV-2 Vaccination Variably Impacts the Antigen-Specific Serologic and T cell Responses

**Figure 3:**
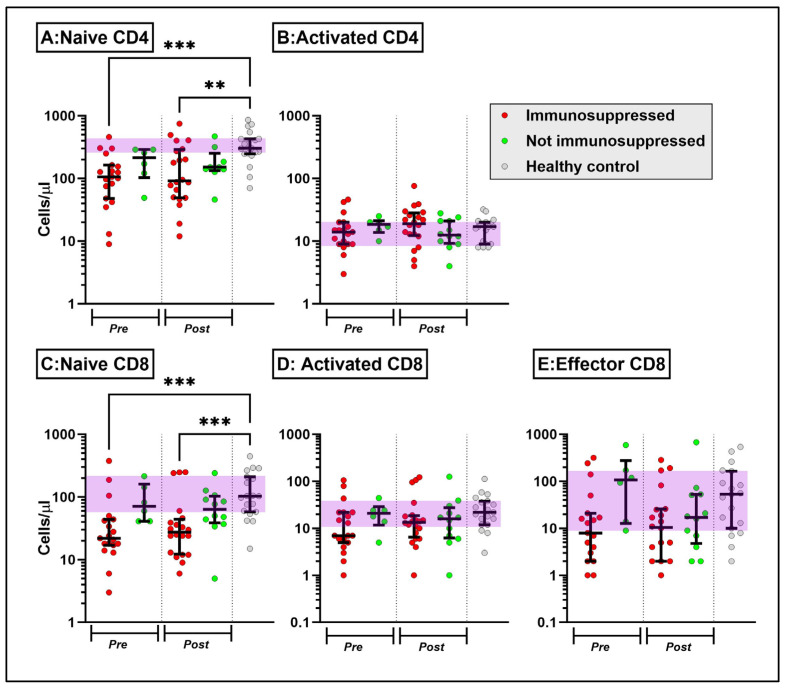
Naïve CD4 (**A**), activated CD4 (**B**), naïve CD8 (**C**), activated CD8 (**D**) and effector CD8 (**E**) lymphocyte subsets in patients with AAV pre- and post-SARS-CoV-2 vaccination, stratified according to the presence or absence of immunosuppression at the time of vaccination (median ± interquartile range). The purple shading represents the interquartile range of healthy uninfected controls. ** *p* < 0.005, and *** *p* < 0.001, Kruskal–Wallis test with Dunn’s post hoc test comparing individual groups against healthy controls.

**Figure 4:**
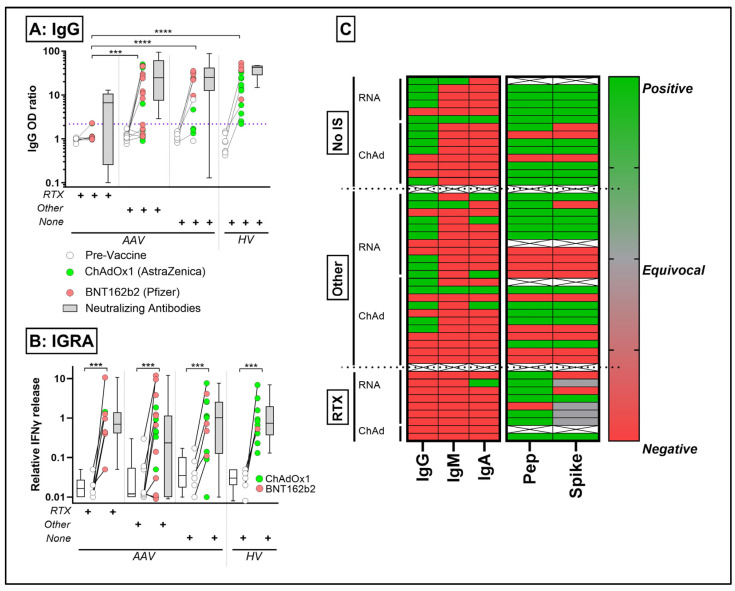
SARS-CoV-2-specific IgG (optical density (OD) ratio of test to irrelevant antigen, (**A**)) and whole blood interferon-γ release assay (COVID-IGRA, (**B**)) in patients vaccinated against SARS-CoV-2. “+” indicated presence of immunosuppressive therapies. The dotted line in (**A**) reflects the 5th centile in the healthy volunteer (HV) group and the grey bars reflect the median and interquartile range of neutralising antibody OD ratio. In (**B**), the white and grey bars reflect the IGRA median and interquartile range. (**C**) summarises the subject level IgG/IgA/IgM and IGRA responses. Pep = spike peptides (S, S1, S+ Peptivator; RTX = Rituximab; Other = other immunosuppression. *** *p* < 0.001, **** *p* < 0.0001 Kruskal–Wallis test with Dunn’s post hoc test comparing individual groups against healthy controls.

We next analysed a separate cohort of patients with AAV who did not have prior COVID-19 and who received two doses of the SARS-CoV-2 vaccine. We first assessed circulating leukocyte subset counts before and after vaccination (Supplemental Tables S1 and S2). Pre-vaccine, immunosuppressed patients with AAV had evidence of NK and T cell cytopenia when compared to healthy controls (Supplemental Figure S3). T cell changes were primarily limited to the naïve CD4 and CD8 subsets (Figure 3) and were not impacted by vaccination. Myeloid subsets were also not significantly impacted by vaccination or immunosuppression (Supplemental Figure S4). All rituximab-treated patients, 41% of those on other immunosuppressants and 28% of non-immunosuppressed patients failed to mount an antigen-specific IgG response (Figure 4A). Conversely, most rituximab-treated patients mounted a strong IFN-γ response to the PepTivator SARS-CoV-2 spike peptide pool at a level similar to healthy controls; 45% of those on other immunosuppressants failed to mount a spike protein IFN-γ response (Figure 4B). Figure 4C summarises the association between immunoglobulin isotypes and IGRA response; notably, few participants mounted an effective IgA or IgM response.

### 2.4. Immunosuppression with B Cell Depleting Agents at the Time of SARS-CoV-2 Vaccination Does Not Alter Non-B Cell Lymphoid Subsets

**Figure 5:**
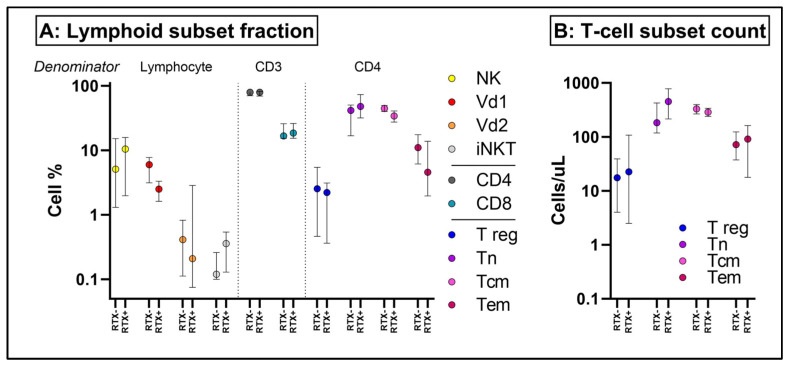
In depth T cell subset analysis of post-vaccine samples in patients exposed to rituximab (RTX+) and those on no immunosuppression (RTX-). (**A**) reflects the cell fraction and (**B**) the absolute cell count (median ± interquartile range). NK = natural killer cell; VD1 = Vδ1 γδ T cell; Vd2 = Vδ2 γδ T cell; iNKT = invariant natural killer T cell; T reg = regulatory T cell; Tn = naïve T cell; Tcm = central memory T cell; Tem = effector memory T cell; no significant differences between exposure groups were observed (mixed effects model with Šídák’s multiple comparisons test).

In view of the disconnect between serologic and cellular immune response to vaccination in rituximab-treated patients, who have been observed to have an overall worse COVID-19 outcome despite vaccination [11], we sought to examine in detail whether Rituximab, in addition to depleting B cells, also indirectly affects other lymphoid subsets (Supplemental Figure S5). As expected, B cells (CD19+CD3-) were profoundly depleted in the Rituximab-treated patients (Supplemental Figure S6), but we did not observe any significant changes in natural killer (CD56+CD3-), conventional, γδ (CD3+ Vδ1+/Vδ2+) or invariant natural killer T cell (Va24Ja18+) populations (Figure 5), nor in monocyte (CD14+CD3-) or dendritic cell (CD3-CD19-CD14- CD11c+/CD1c+/CD1a+/CD123+) populations (Supplemental Figure S6).

## 3. Discussion

In this prospective study of 182 individuals, we demonstrated an altered immune response in acute COVID-19 in those who were exposed to immunosuppressive therapy. We comprehensively described the immunologic responses of patients with AAV who received SARS-CoV-2 vaccination and confirm that a high proportion mount an incomplete response compared to immunocompetent individuals, particularly those exposed to B cell depleting therapy, with the preservation of antigen-specific T cell responses and no alteration in non-B cell lymphoid subsets in these patients.

Lymphopenia is an important clinical factor that is significantly and independently associated with a higher risk of poor outcomes in SARS-CoV-2 infection [12,13]. Immunosuppressed participants had reduced naïve CD4/CD8 cell counts, but only naïve CD4 cells remained low in these patients into convalescence. The CD8 response in non-immunosuppressed patients seen in our cohort is typical of COVID-19 [14,15], but this effect was blunted in those receiving immunosuppressive therapies. Lymphopenia in AAV can be a risk factor for severe infections [16], an effect that was worsened by longer duration of corticosteroid use. The increase in activated CD4 and CD8 cells seen in the non-immunosuppressed group was not observed in the immunosuppressed patient population, suggesting an abrogation of the COVID-19 hyperimmune response in this group.

A decrease in CD16+ monocyte subsets has been reported in severe COVID-19 [17]. A proportionate decrease in non-classical monocytes occurred that appeared more marked in immunosuppressed patients. This suggests that the innate immune response triggered by SARS-CoV-2 infection suppresses the immunosuppressed patients’ already dysfunctional immune system even further [18]. Previous work from our group has shown expansion of the intermediate monocyte subset in AAV subtypes [19]. These cells also produce more proinflammatory cytokines than other cell types, which may contribute to increased circulating cytokines in these patients. We have also explored the similar disruption of neutrophil subsets in AAV [20], where immature neutrophils are enriched. These increases in immature neutrophil levels have been reported in severe SARS-CoV-2 infection [17,18], but were not seen in our immunosuppressed population.

Comparing AAV patients before and after COVID-19 vaccination, naïve CD4 and CD8 T cells remain persistently low in the immunosuppressed groups both before and after vaccination compared to healthy controls (HC, Figure 3A,C).

Following COVID-19 vaccination, 72% of non-immunosuppressed and 59% of immunosuppressed AAV patients were able to mount an antigen-specific IgG response (Figure 4A). None of the patients taking the B cell-depleting therapy rituximab showed a detectible serological response, in line with results from similar cohorts. While persistent lymphopenia may predispose these patients to greater risk of severe infections [16], the T cell response was adequate in this group. In addition, Further deep phenotyping did not reveal any novel differences in T cell subsets between rituximab- and other immunosuppression-exposed groups. However, the activation of other T cell types may be reliant on additional leukocyte crosstalk [21].

A recent meta-analysis found that, compared with immunocompetent controls, the pooled risk ratios for seroconversion after a first vaccine dose was 0.53 in those with immune mediated inflammatory disorders, increasing to 0.75 after the second dose [22]. Prendecki et al. reported that 59.3% of fully vaccinated AAV patients seroconverted [11], but that those taking rituximab reduced antibody responses compared to other treatments. Similarly, an impaired serological response has also been observed in other patient cohorts receiving anti-CD20-targeting therapies [23,24]. In the OCTAVE study, antibody responses remained low after two doses of the vaccine [9], and in the MELODY study, participants with plasma cell malignancies who had received anti-CD20 therapies in the preceding 3 months were least likely to have a positive antibody response (OR 0.05 [0.04–0.06]). Looking at disease subtypes among the autoimmune rheumatic disease cohort in the MELODY study, those with AAV were least likely to have a positive antibody response (OR 0.69 [0.55–0.87]) [10]. Concurrent corticosteroid use was also associated with reduced antibody response, independent of other immunosuppression [10]. Our results confirm that AAV patients on B cell targeted therapies were unable to mount an effective antibody response to SARS-CoV-2, but other immunosuppressive regimens had a less pronounced effect.

Our data show that following COVID-19 vaccination, the T cell response appears to be less impacted than the humoral (B cell) response. Most (87.5%) rituximab-treated patients mounted a strong IFN-γ response to the PepTivator SARS-CoV-2 spike peptide pool at a level similar to healthy controls. However, only 55% of those on other immunosuppressants had a sufficient IFN-γ response. In the OCTAVE study, T cell responses were strong after a single dose in the AAV group receiving B cell-depleting therapies [6], and a UK cohort reported an 82.6% T cell response rate in immunosuppressed individuals after two vaccine doses [11].

Here we show that, even in the absence of a sufficient humoral/antibody response in rituximab-exposed individuals, AAV patients’ T cells maintained their ability to produce IFN-γ in response to SARS-CoV-2 antigens. IFN-γ promotes antiviral immunity, acting as a key link between the innate immune response and activation of adaptive immune response like that of Type 1 interferon [25]. Seree-Aphinan et al. showed that in those receiving rituximab therapy, nine months of rituximab-to-vaccination interval maximises the positive likelihood of seroconversion when compared to those who are rituximab-naïve [26]. This time frame coincided with repopulation of naïve B cells in our patients. This study also showed that increasing the time interval of rituximab-to-vaccination to 12 months did not produce a significant increase in seroconversion rate [26]. Smith et al. showed that a single SARS-CoV-2 vaccine dose given more than 6 months after the last dose of rituximab treatment and receiving a mRNA vaccine as compared to adenovirus vector vaccine were independently associated with lower risk of hospitalisation for COVID-19. The receipt of an additional booster dose of vaccine was also associated with decreased risk of hospitalisation for COVID-19 [27]. Therefore, vaccination against SARS-CoV-2 infection in patients receiving rituximab infusion should be performed no less than 6 months and ideally at 9 months after their last rituximab infusion but should not be prolonged beyond this in order to reduce the risk of infection.

This study was conducted prospectively within the framework of a national registry and is an excellent example of how these resources can be quickly mobilised to conduct clinical research. We comprehensively describe the clinical characteristics, immunologic responses, and clinical outcomes of SARS-CoV-2 infection and vaccination in immunosuppressed patients. In particular, this study examines these conditions in AAV patients and builds on previous work showing incomplete response to vaccination, relative to immunocompetent individuals [11]. Our study also has several limitations worth noting. Virtually all participants were of a white, northern European background, limiting generalisability to other populations. Several patients were unable to be sampled at the time of SARS-CoV-2 infection or prior to receiving their vaccination. Finally, the T cell response assay is an assay measuring IFN-γ production in whole blood following stimulation. While this is an effective estimate of T cell reactivity, it is possible that small amounts of IFN-γ were produced by other cell types. Our findings may directly inform clinical decision-making with respect to the choice, timing, and frequency of SARS-CoV-2 vaccination and approaches to immunosuppressive therapy management in the setting of acute COVID-19 infection and/or planned vaccination in this vulnerable patient population.

## 4. Materials and Methods

### 4.1. Study Cohorts

Two cohorts were studied: one focusing on acute COVID-19 (Cohort 1, Table 1) and one on SARS-CoV-2 vaccination (Cohort 2, Table 2). Participants were either AAV patients previously recruited to a national vasculitis registry, the RITA Ireland Vasculitis (RIV) Registry and Biobank [28], or participants recruited to the St James’s, Tallaght University Hospital, Trinity Alliance for Research (STTAR) COVID-19 Biobank [29]. The STTAR Bioresource study was granted ethical approval by the SJH/TUH Joint Research and Ethics Committee (JREC 2020-05 List 19). Prior ethical approval was obtained from AAV patients in all RIV recruiting sites. This project received additional approval from the St. James Hospital/Tallaght Ethics Committee (REC: 2020-04 List 13—Amendment 22 [RIV] and JREC 2020-05 List 19 [STTAR]). Longitudinal clinical data were collected and managed using the REDCAP electronic data capture tools hosted at Trinity College Dublin [30], using an eCRF co-developed with the UK and Ireland Vasculitis Society and interoperable with the EULAR/Global Rheumatology Alliance partnership registry.

Cohort 1 comprised two distinct groups during acute COVID-19: patients with rheumatic disease receiving immunosuppressant therapy and patients not receiving immunosuppressant therapy. All participants were unvaccinated and recruited between March 2020 and February 2021, and were thus contemporaneous with the alpha and beta virus variants in Ireland (pre-Omicron B.1.1.529) [31]. Patients were sampled at the time of COVID-19 diagnosis (acute), and again approximately 3 months after infection (convalescent). Cohort 2 comprised patients with AAV (on and off immunosuppression) and healthy controls, pre-vaccination samples were collected between March and June 2021; all vaccine recipients were negative for membrane and nucleocapsid antibodies, indicating that they had no recent history of COVID-19. Paired post-vaccine samples were obtained 6 weeks after the second vaccine dose (median = 42 days; IQR = 35–57). A cohort of SARS-CoV-2 infection- and vaccine-naïve healthy volunteers (HC) served as a control group for comparison of flow cytometry data.

### 4.2. Immunosuppression and Vaccine Exposure

Upon recruitment to the study, patients were defined as rituximab-exposed, immunosuppression-exposed, or non-immunosuppressed based on their treatment history. The rituximab-exposed group included individuals receiving rituximab within the previous 6 months and/or having persistent B cell count < 10 cells/µL. The ‘Other’ immunosuppression-exposed group included individuals exposed to any of the following: cyclophosphamide within the previous 3 months, current azathioprine, mycophenolate mofetil or methotrexate use, and/or current use of prednisolone (or equivalent) ≥ 10 mg daily. Those not currently receiving immunosuppression or receiving < 10 mg prednisolone daily were classed as not exposed to immunosuppression. We categorised SARS-CoV-2 vaccines into either mRNA vaccine (Pfizer/BioNTech [BNT162b2] or Moderna [CX-024414]) or adenovirus-based vaccine (AstraZeneca [AZD1222]).

### 4.3. Immunophenotyping

Immunophenotyping on fresh whole blood samples was carried out as described by Townsend et al. [32]. Briefly, 3–5 mL of blood was collected in plasma-EDTA tubes mid- and post-COVID-19 infection or pre- and post-SARS-CoV-2 vaccination. Cells were acquired using the FACSCanto II flow cytometer using FACSDIVA V8 acquisition and FlowJo V10 analysis software (BD Biosciences, San Jose, CA, USA). Absolute cell counts and relative proportions of T cells (CD3+, CD4+, and CD8+), B cells (CD19+) and NK cells (CD16+CD56+) were calculated using the absolute frequencies of parent populations acquired from the TruCount™ tubes (BD Biosciences, San Jose, CA, USA). Naïve and effector CD4+ or CD8+ T cells were characterised for the expression of CD27, CD45RA, and CD197 in an additional panel. T cell activation was determined by CD38 and HLA-DR expression. Lymphocyte subsets were defined as naïve CD4+ (CD3+CD4+CD45RA+CD27+), naïve CD8+ (CD3+CD8+CD45RA+CD27+CD197+), effector CD8+ (CD3+CD4-CD45RA+CD27-CD197-), activated CD4+ (CD3+CD4+HLADR+CD38+), and activated CD8+ (CD3+CD8+HLADR+CD38+). T cell phenotyping assays were validated and accredited in line with ISO15189 standards [33]. Classical, intermediate, and non-classical monocytes were characterised by levels of CD14 and CD16 expression. CD10 and CD16 expression by neutrophils was also determined.

To assess the in-depth lymphoid cell immunophenotype post-vaccination in rituximab-treated patients, we used cryopreserved peripheral blood mononuclear cells (PBMC), acquired using the Aurora full spectrum cytometer (Cytek Biosciences, Fremont, CA) across two staining panels (Supplementary Tables S3 and S4). The first panel (P1) primarily characterised T cell subsets with the second panel (P2) characterising other principal immune cell populations within the PBMC sample.

### 4.4. T Cell Responses

An interferon gamma response assay (IGRA) was used to approximate T cell reactivity to SARS-CoV-2 [34]. A total of 5 mL of blood was collected in a lithium heparin tube and processed within 4 h of collection. 1 mL of blood was decanted into blood monitor tubes and relevant stimulants were added: unstimulated/null (negative control); lyosphere (anti-CD3 & R848, positive control); and peptide pools (Miltenyi Biotec, Bergisch Gladbach, Germany) consisting of spikes (S, S1, and S+; 0.5 μg/mL), membranes, and nucleocapsid (0.5 μg/mL) and a recombinant spike protein trimer (amino-acids 14-1213, 1 μg/mL). Tubes were incubated for 24 h at 37 °C then centrifuged at 2000–3000 g for 15 min. Interferon gamma (IFN-ɣ) production was measured by ELISA (Qiagen, Manchester, UK). Qiagen QuantiFERON^®^ Monitor ELISA kit was used to measure IFN-ɣ levels. Results are reported as IU/mL in response to stimulus, subtracting the ‘Unstimulated/Null’ value from the ‘Stimulated’ value. The reference range for IFN-ɣ response to spike peptides (S, S1, S+) was 0.11 IU/mL. This range was established using receiver operator characteristic curves for 30 healthy controls pre- and post-vaccination.

### 4.5. Serology

A novel serology assay developed by Phelan et al. [35] was used to measure IgG, IgM, and IgA antibody levels in pre- and post-vaccination samples. Briefly, 96-well plates (Greiner Bio-one, Kremsmünster, Austria, #655061) were coated with 50 µL of 2 µg/mL SARS-CoV-2 RBD or full-length spike trimer in PBS (Gibco, Cork, Ireland #10010015) and left overnight at 4 °C. The following morning, the coating solution was removed and 100 µL of 3% non-fat milk (Marvel, Hertfordshire, United Kingdom), prepared in PBS with 0.1% Tween 20 (PBST), was added to each well as blocking buffer. Serum samples were diluted 1:50 in 1% milk PBST, and 100 µL was added to wells in triplicate and left for 2 h at room temperature. The HRP-conjugated secondary antibodies were diluted in 1% milk PBST and 100 µL/well was added for 1 h at room temperature; IgG1 (1:2000, Southern Biotech, Birmingham, AL, USA, #9054-05), IgM (1:3000, Merck, Cork, Ireland #A6907-1ML), and IgA (1:3000, Merck, Cork, Ireland #A0295-1ML). The antibody solution was removed, and the plates were again washed three times with 0.1% PBST. A total of 100 µL SigmaFast OPD (o-phenylenediamine dihydrochloride, Merck, #P9187-50SET) was added to each well for 10 min, and the reaction was stopped using 3 M hydrochloric acid. Cut-off values were determined by averaging the OD values for 103 pre-COVID-19 serum samples plus the addition of 3 standard deviations. Levels of serum IL-6 and soluble CD25 were established in selected samples using the ELLA ProteinSimple Multiplex ELISA assay (BioTechne, Minneapolis, MN, USA).

### 4.6. Statistical Methodology

Assay readouts are summarised as median and interquartile range unless otherwise advised. The overall difference between groups in each of the flow cytometry studies was analysed with a non-parametric Kruskal–Wallis test, with Dunn’s multiple comparison post hoc test used to assess the difference between individual groups and a healthy control cohort. To assess the impact of immunosuppression on serological and IGRA responses to vaccination, and the impact of rituximab on lymphocyte subset phenotype, we used a mixed effects analysis with Tukey’s multiple comparisons test.

## 5. Conclusions

Firstly, our study confirms findings from international cohorts of immunosuppressed patients (with autoimmune or rheumatic disease, cancer, or organ transplantation) in an Irish cohort of patients with vasculitis. Furthermore, we comprehensively describe the immunologic responses in immunosuppressed patients who experienced acute COVID-19 or received SARS-CoV-2 vaccination and confirm that a high proportion fail to mount an adequate IgG antibody response, particularly those exposed to B cell depleting therapy, despite T cell responses that were comparable to those of their non-immunosuppressed counterparts.

## Figures and Tables

**Table 1:** Baseline-characteristics of Cohort 1 (acute SARS-CoV-2 cohort). Also shown is the comparator healthy control (HC) cohort.

		SARS-CoV-2-Infected		SARS-CoV-2-Naïve
		Immunosuppressed	Non-Immunosuppressed	Healthy Controls
Population (n = 102)	N (%)	19 (18.6%)	83 (81.4%)	19
Sex N (%)	Male	8 (42.1%)	43 (51.8%)	7 (36.9%)
	Female	11 (57.9%)	40 (48.2%)	12 (63.1%)
Age	Median (IQR)	55 (46–70)	62 (52–72)	48 (26–79)
Immunosuppressed N (%)	Rituximab	3 (2.9%)	-	-
	Other	16 (15.7%)	-	-
	None	-	83 (81.4%)	19 (100.0%)

**Table 2:** Baseline patient characteristics at time of first SARS-CoV-2 vaccination in patients with AAV (Cohort 2). Also shown is the comparator healthy control (HC) cohort. NA = not available.

		AAV	Immunosuppressed	Non-Immunosuppressed	HC
Population N (%)		43	28 (65.1%)	15 (34.9%)	18
Sex N (%)	Male	24	17 (60.7%)	7 (46.7%)	8
	Female	19	11 (39.3%)	8 (53.3%)	10
Age	Median (IQR)	59.5 (46–78)	67.5 (63–74)	62 (53–67)	41.5 (36–52)
Smoking N (%)	Current	0	0 (0.0%)	1 (0.0%)	NA
	Past	15	9 (32.1%)	6 (40.0%)	NA
	Never	13	7 (25.0%)	6 (40.0%)	NA
	NA	15	12 (42.9%)	3 (20.0%)	NA
Vaccine Type N (%)	AstraZeneca	18	10 (35.7%)	8 (53.3%)	13
	Pfizer/BioNTech	22	16 (57.1%)	6 (40.0%)	5
	Moderna	3	2 (7.1%)	1 (6.7%)	0
Vaccine Interval (Days)	Median (IQR)	28 (28–37)	28 (28–35)	28 (28–37)	NA
Immunosuppression Type N (%)	Rituximab	7	7 (25.0%)	-	0
	Other	21	21 (75.0%)	-	0
	None	15	-	15 (100.0%)	18
	NA	1	-	-	-

## Data Availability

The original flow cytometry data presented in the study are openly available on the Zenodo platform.

## Supplementary Materials

The following supporting information can be downloaded at: https://www.mdpi.com/article/10.3390/ijms25105239/s1.
