# Supplementary material for: Effect of Immunosuppression on the Immune Response to SARS-CoV-2 Infection and Vaccination
Source: Int J Mol Sci. 2024 May 11;25(10):5239. doi: 10.3390/ijms25105239 (PMC11120762; doi:10.3390/ijms25105239)
Supplement: Supplementary file 1 [file ijms-25-05239-s001.zip › ijms-2972345-supplementary.pdf]

Supplemental Material

Contents

Supplemental Material 1

Supplemental Figure S1. T/B/NK cell counts during and after COVID-19........ 2

Supplemental Figure S2. IL6 and sCD25 levels........ 3

Supplemental Figure S3. T/B/NK cell counts pre and post vaccination........ 4

Supplemental Figure S4. Myeloid cells pre and post vaccination 5

Supplemental Figure S5. CD4 T cell fractions in vaccinated participants stratified by rituximab (RTX) exposure status. 6

Supplemental Figure S6. B-cell and myeloid cell subsets in vaccinated participants stratified by rituximab (RTX) exposure status........ 7

Supplemental Table S1. Lymphocyte fractions and absolute cell counts in participants with acute COVID-19, stratified by immunosuppression status........ 8

Supplemental Table S2. Lymphocyte fractions and absolute cell counts in vaccinated participants, stratified by immunosuppression status. 9

Supplemental Table S3. Immunophenotyping panel 1........ 10

Supplemental Table S4. Immunophenotyping panel 2........ 11

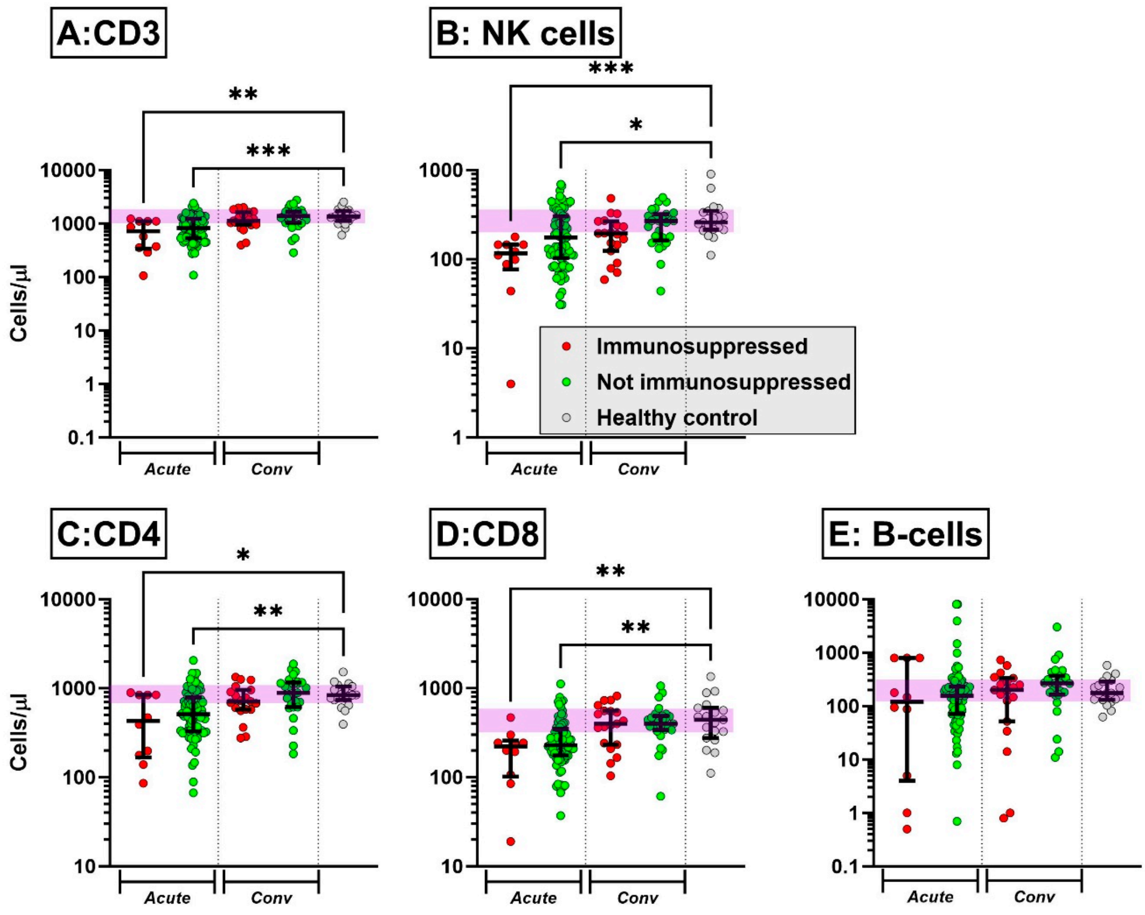

Supplemental Figure S1. T/B/NK cell counts during and after COVID-19

Absolute circulating CD3+ (A), NK cell (B), CD4+ (C), CD8+ (D) and B-cell counts (median ± interquartile range) in patients with acute COVID (Acute) and 3 months post infection (Conv), stratified according to the presence of immunosuppression at the time of infection. The pink shading represents the interquartile range of healthy uninfected controls. *p<0.5, **p<0.005, ***p<0.001, Kruskal Wallis test with Dunn's post hoc test comparing individual groups against healthy controls.

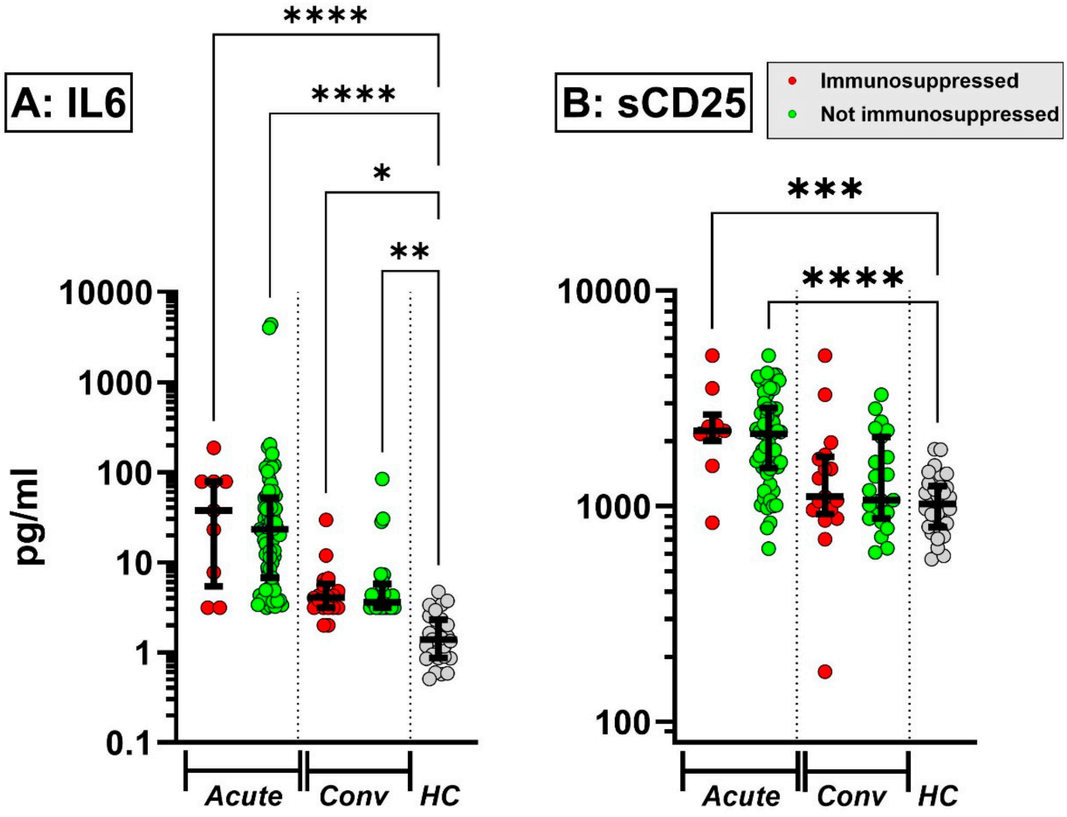

Supplemental Figure S2. IL6 and sCD25 levels

Serum IL6 (A) and sCD25 (B) levels in participants on immunosuppression (red) and those not receiving immunosuppression (green), during SARS-CoV2 infection and in the convalescent period. Kruskal Wallis test with Dunn's post hoc test comparing individual groups against healthy controls (HC) in A. HC data was not available for sCD25 so immunosuppressed and non-immunosuppressed were compared directly. The pink shading represents the clinical laboratory normal reference range.

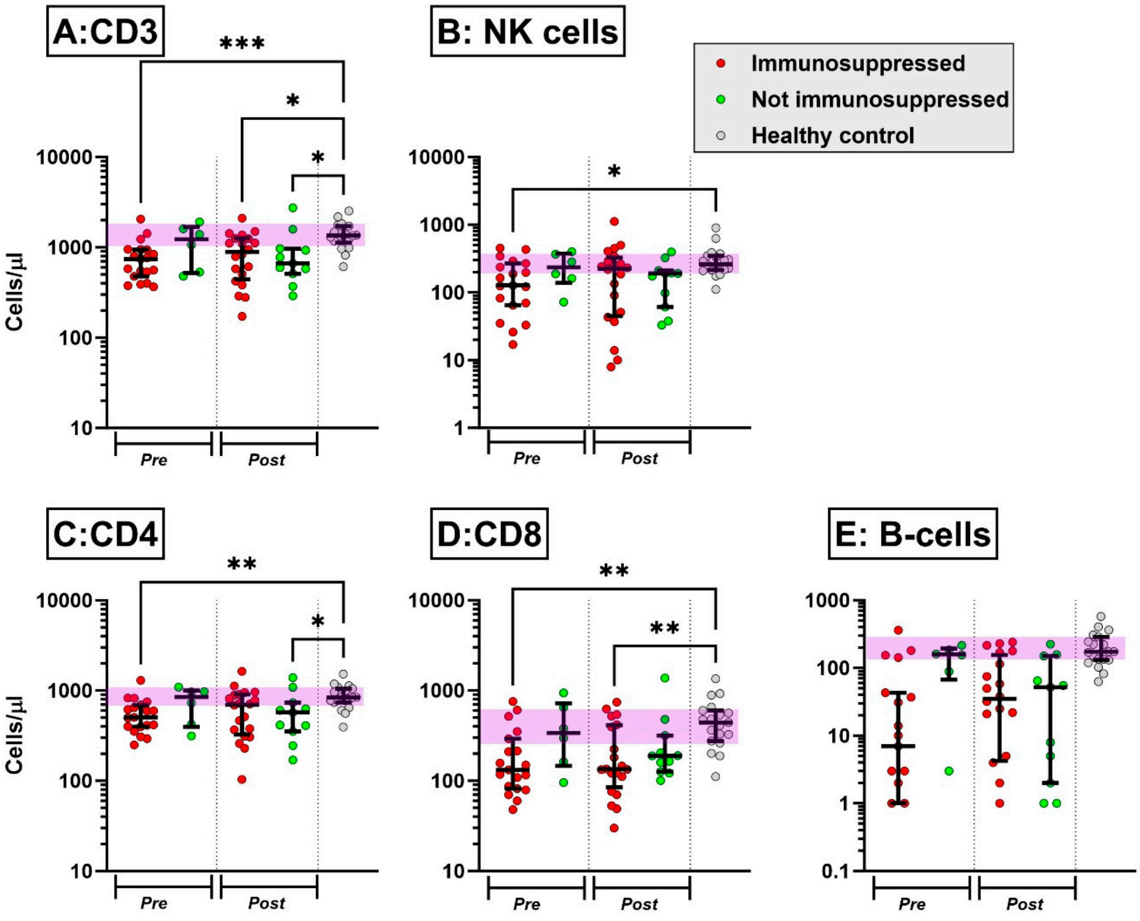

Supplemental Figure S3. T/B/NK cell counts pre and post vaccination.

Absolute circulating CD3+ (A), NK cell (B), CD4+ (C), CD8+ (D) and B-cell counts (median ± interquartile range) pre and post vaccination, stratified according to the presence of immunosuppression at the time of vaccination. The pink shading represents the interquartile range of healthy uninfected controls. *p<0.5, **p<0.005, ***p<0.001, Kruskal Wallis test with Dunn's post hoc test comparing individual groups against healthy controls.

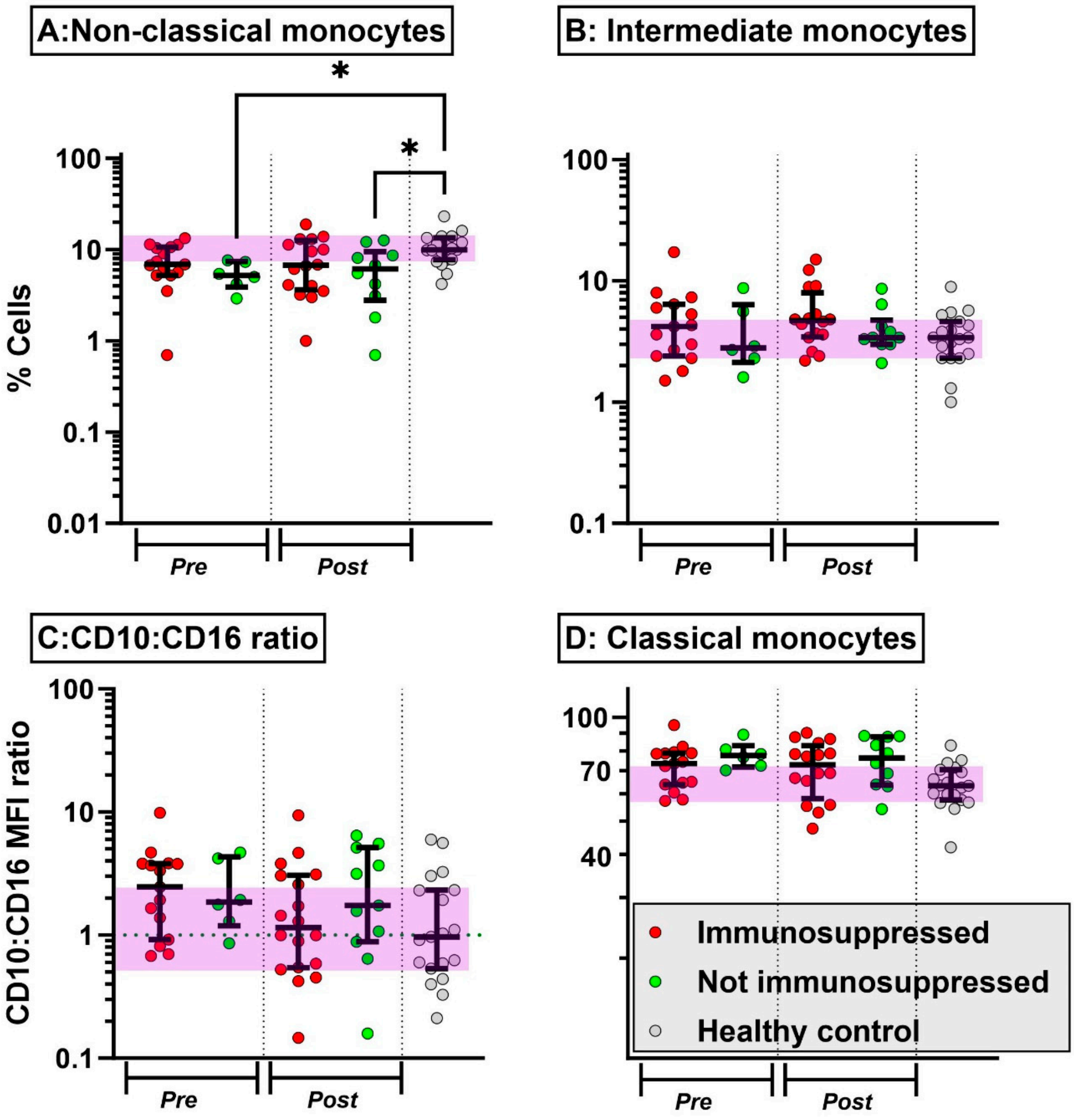

Supplemental Figure S4. Myeloid cells pre and post vaccination

Fraction of non-classical (A), intermediate (B) and classical (D) monocytes, and the ratio of CD:cD16 on neutrophils (C) (median ± interquartile range) before and after receiving a SARS-CoV2 vaccine, stratified according to the presence of immunosuppression at the time of infection. The pink shading represents the interquartile range of healthy uninfected controls. *p<0.5, Kruskal Wallis test with Dunn's post hoc test comparing individual groups against healthy controls.

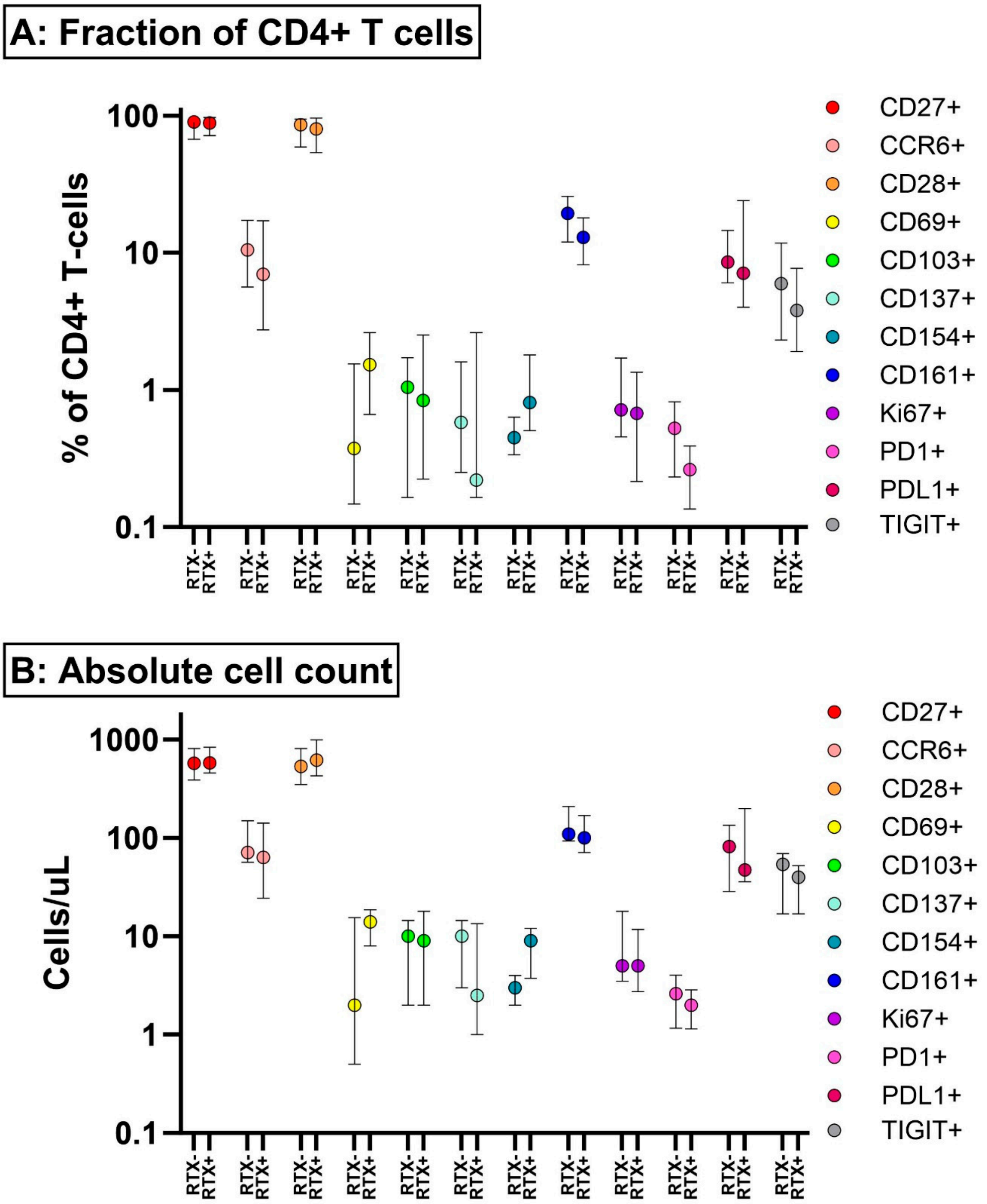

Supplemental Figure S5. CD4 T cell fractions in vaccinated participants stratified by rituximab (RTX) exposure status.

Fraction (A) and absolute cell count (B). All plots represent median and interquartile range. No significant differences were observed between the RTX exposure groups.

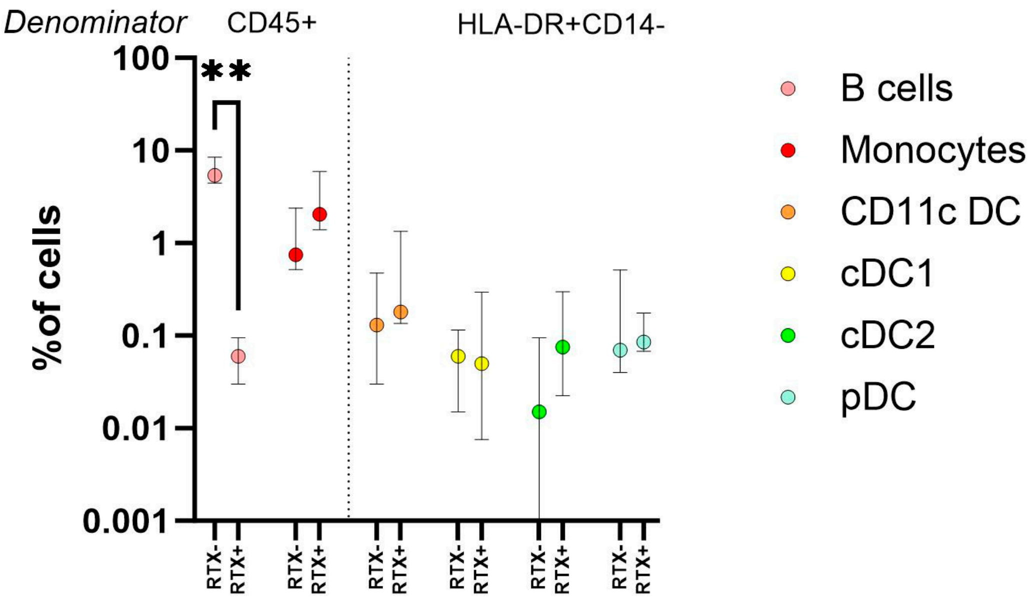

Supplemental Figure S6. B-cell and myeloid cell subsets in vaccinated participants stratified by rituximab (RTX) exposure status.

Fraction (A) and absolute cell count (B). All plots represent median and interquartile range. 2-way ANOVA with Sidak's multiple comparisons test, **p<0.005.

	Acute Covid		Convalescent	
Cell fraction	**Immunosuppressed**	**No IS**	**Immunosuppressed**	**No IS**
**CD3 %**	80 (66.5-82)	69 (61-75.8)	74 (71-82)	70 (56.5-72)
**CD4 %**	47 (40.5-56)	47 (37-54)	47 (43-54)	42 (31.5-50.5)
**CD8 %**	24 (16-34)	19 (15-26.5)	29 (24-31)	15 (14.5-21.5)
**B cell %**	10 (4.5-18)	13 (6-18)	12 (3-19)	13 (8.5-28.5)
**NK %**	8 (6.5-15)	14 (9-22)	12 (8-15)	13 (9-20.5)
**NaiveCD4 %**	37.5 (28.15-49.85)	37.2 (23-45.1)	20.1 (15.6-23.8)	22.5 (7.6-36)
**NaiveCD8 %**	31.2 (17.6-43.7)	19.5 (8.4-30.3)	20.9 (6.5-26.1)	4.8 (4.2-25.25)
**EffectorCD8 %**	7.6 (3.55-29.05)	21.1 (9.8-36.2)	10 (4.7-27.7)	21.9 (4.85-29.5)
**Activated CD4%**	2.6 (1.65-7.35)	4.2 (2.5-7.1)	2.5 (1.7-4.6)	2.7 (1.65-4.75)
**Activated CD8%**	16.7 (8.45-24.55)	19.3 (11.6-34)	7.4 (5.2-12.4)	18.3 (8-29.45)

	Acute Covid		Convalescent	
Absolute cell count	**Immunosuppressed**	**No IS**	**Immunosuppressed**	**No IS**
**Count.CD3**	861 (475-1223)	834 (548-1228.5)	1058 (958-1491)	1034 (970-1113)
**Count.CD4**	604 (285-869.5)	519 (332-792.5)	677 (578-788)	702 (608-824.5)
**Count.CD8**	243 (196.5-489.5)	228 (177-357.5)	399 (360-645)	293 (237.5-370.5)
**Count.B**	147 (46.5-242.5)	156 (79-232)	193 (34-334)	183 (132.5-579.5)
**Count.NK**	122 (89.5-157.5)	175 (103-292.5)	196 (79-267)	197 (169-252)
**Naïve CD4.count**	176 (54-418.5)	204 (89-326)	151 (98-172)	205 (51.5-256.5)
**Naïve CD8.count**	73 (40-110.5)	39 (17-77)	56 (29-112)	20 (15.5-59)
**Effector CD8.count**	33 (7.5-103)	44 (12-79)	51 (10-144)	86 (11.5-105.5)
**Activated CD4 count**	12 (11-19)	24 (14-39)	15 (12-28)	19 (14.5-34)
**Activated CD8 count**	32 (19-59)	50 (22-92)	28 (13-61)	37 (25.5-101)

Supplemental Table S1. Lymphocyte fractions and absolute cell counts in participants with acute COVID-19, stratified by immunosuppression status.

Values represent median (interquartile range).

	Pre-Vaccine			Post-Vaccine		
Cell fraction	**Rituximab**	**Other IS**	**No IS**	**Rituximab**	**Other**	**No IS**
**CD3 %**	81 (76.5-87.5)	83 (61.5-89)	69 (61-82)	78 (72-84.5)	76 (69-87)	75.5 (69.25-83.25)
**CD4 %**	63 (57.5-65)	53 (48-67)	45 (43-49)	59 (52-64.5)	55 (46-65)	52 (43-61.25)
**CD8 %**	18 (12.5-26.5)	13 (11-24)	23 (17-25)	19 (14.5-28)	14 (10-23)	23 (19-27)
**B cell %**	0 (0-0)	5 (1.5-13)	12 (8-14)	0 (0-0.5)	11 (3-12)	7.5 (2.75-10.75)
**NK %**	17 (11.5-21.5)	10 (6.5-23.5)	18 (13-22)	21 (14-23.5)	12 (7-19)	17 (9.75-18.75)
**NaiveCD4 %**	31 (21-43.6)	16.3 (12.15-24.25)	28.6 (23.5-29.8)	26.6 (18.1-42.15)	20 (12.1-33.6)	25.1 (20.65-31.55)
**NaiveCD8 %**	22.6 (20.05-33.45)	21.9 (10.55-33.2)	20.7 (15.2-25)	21.3 (11.3-45.2)	26.7 (15.2-39.7)	25.2 (17.7-36)
**EffectorCD8 %**	5.9 (3.65-27.75)	3.1 (1.55-10.55)	31.2 (25.3-42)	14.2 (5.25-21.2)	3.1 (2.6-4.9)	11.2 (5.35-26.4)
**Activated CD4%**	2.3 (1.75-2.7)	3 (1.8-4.1)	2.3 (1.9-2.7)	2.8 (1.8-3.55)	3.4 (2.6-4.3)	2 (1.8-2.6)
**Activated CD8%**	6 (5-8.55)	7.2 (4.3-10.55)	6.6 (4.9-8)	6.1 (3.5-11.45)	9.6 (4.3-11.5)	9.1 (7.1-10.65)

	Pre-Vaccine			Post-Vaccine		
Absolute cell count	**Rituximab**	**Other IS**	**No IS**	**Rituximab**	**Other IS**	**No IS**
**Count.CD3**	944 (673.5-949.5)	560 (467.5-819.5)	1069 (532-1604)	1121 (883-1345.5)	579 (384-1112)	959 (629.5-1469)
**Count.CD4**	599 (459-700.5)	505 (377-656.5)	729 (424-976)	728 (641-850.5)	466 (306-784)	617 (463-1042)
**Count.CD8**	211 (100-322)	116 (78.5-186)	306 (163-649)	395 (170-469.5)	121 (70-146)	253.5 (183-442.75)
**Count.B**	0 (0-1)	37 (12-148.5)	162 (116-186)	2 (0.5-4.5)	50 (25-179)	113 (57.5-163.25)
**Count.NK**	127 (123-218)	164 (34-279.5)	242 (161-367)	211 (190.5-315.5)	134 (37-264)	224.5 (178.25-311.75)
**NaiveCD4 count**	164 (128-276)	97 (58.5-116.5)	207 (121-260)	196 (113-383.5)	78 (49-178)	185 (147-244.5)
**NaiveCD8 count**	42 (18-74.5)	22 (16-31)	60 (41-82)	34 (24-142)	30 (12-36)	77 (49-88)
**EffectorCD8 count**	13 (4-78.5)	7 (1.5-16)	119 (36-166)	26 (11.5-126.5)	5 (2-11)	21 (12.5-104)
**Activated CD4 count**	15 (9.5-15)	13 (9-20)	17 (12-20)	20 (11.5-22.5)	18 (12-26)	15 (11-22.5)
**Activated CD8 count**	7 (6.5-29)	7 (4.5-22)	24 (14-35)	11 (7-62.5)	12 (6-15)	21 (14-40.5)

Supplemental Table S2. Lymphocyte fractions and absolute cell counts in vaccinated participants, stratified by immunosuppression status.

Values represent median (interquartile range).

Panel 1		
**Target**	**Fluorochrome**	**Clone**
Vα24Jα18	BV421	6B11
CD154	SB436	TRAP1
Vδ2	VioBlue	REA771
Vα7.2	BV480	OF-5A12
CD69	BV510	FN50
CD56	BV570	HCD56
CD137	BV605	6H6
Ki67	BV650	B56
CD28	BV711	CD28.2
CCR7	BV750	3D12
TIGIT	BV786	741182
CD27	BB515	M-T271
CD3	Spark Blue 550	SK7
CD8	PerCP	SK1
CD4	BB700	SK3
CD103	PerCP-eF710	Ber-ACT8
Vδ1	PE	TS8.2
CD57	PE-CF594	NK-1
CD25	PE-Fire640	M-A251
CD161	PE-Cy5	DX12
CD45RA	PE-Cy5.5	HI100
CCR6	PE-Cy7	R6H1
PD-1	AF647	MIH4
PD-L1	APC	MIH1
CD127	Spark NIR 685	AO19D5
FoxP3	R718	259D/C7
CD45	APC-Fire 810	Hi30

Supplemental Table S3. Immunophenotyping panel 1

Panel 2		
**Target**	**Fluorochrome**	**Clone**
CD11c	BV421	3.9
CD1a	BV480	HI149
CD19	BV510	HIB19
CD14	BV570	M5E2
IgD	BV605	δ-IA6-2
CD20	BV650	2H7
CD141	BV711	1A4
CD24	BV786	ML5
CD1c	BB515	F10/21A3
CD3	Spark Blue 550	SK7
CD38	BB700	HIT2
CD16	PE	3G8
CD138	PE-CF594	MI15
CD161	PE-Cy5	DX12
HLA-DR	PE-Cy7	G46-6
CD64	APC	10.1
CD127	SparkNIR 685	AO19D5
CD123	AF700	6H6
CD45	APC-Fire 810	Hi30
N/A	Zombie NIR	N/A

Supplemental Table S4. Immunophenotyping panel 2
